# Effect of Palm Oil–Carnauba Wax Oleogel That Processed with Ultrasonication on the Physicochemical Properties of Salted Duck Egg White Fortified Instant Noodles

**DOI:** 10.3390/gels8080487

**Published:** 2022-08-05

**Authors:** Paramee Noonim, Bharathipriya Rajasekaran, Karthikeyan Venkatachalam

**Affiliations:** 1 Faculty of Innovative Agriculture and Fishery Establishment Project, Prince of Songkla University, Surat Thani Campus, Makham Tia, Muang, Surat Thani 84000, Thailand; 2 International Center of Excellence in Seafood Science and Innovation, Faculty of Agro-Industry, Prince of Songkla University, Hat Yai, Songkhla 90110, Thailand

**Keywords:** palm oil, oleogel, carnauba wax, ultrasonication, salted duck egg white, Chaiya, instant noodles, physicochemical properties, microstructure, storage stability

## Abstract

The present study permutes edible palm oil (PO) into oleogel by incorporating carnauba wax (CW) at two different concentrations (5 g/100 g and 10 g/100 g, *w*/*w*) and processing using ultrasonication. The prepared oleogels (OG1: PO-CW (5 g/100 g); OG2: PO-CW (10 g/100 g); and OGU1: PO-CW (5 g/100 g) with ultrasonication, and OGU2: PO-CW (10 g/100 g) with ultrasonication) were compared with PO (control) to deep fry salted duck egg white (SDEW) fortified instant noodles. The impact of different frying mediums on the physicochemical properties of SDEW noodles was investigated. SDEW instant noodles that were fried using OGU and OG samples had a higher L* and b* but lower a* values than those that were fried in PO (*p* < 0.05). Among the oleogel-fried samples, noodles that were fried in OGU2 and OG2 effectively lowered the oil uptake and showed better cooking properties than OGU1- and OG1-fried noodles, respectively (*p* < 0.05). Textural attributes such as higher hardness, firmness, chewiness, tensile strength and elasticity, and lower stickiness were noticed in the samples that were fried in OGU, followed by OG and PO (*p* < 0.05). Scanning electron microstructure revealed a uniform and smoother surface of noodles fried in OGU and OG, whereas the PO-fried sample showed an uneven and rough surface with more bulges. Noodles were tested for fatty acid compositions, and the results found that oleogel-fried noodles retained more unsaturated fatty acids than the control (*p* < 0.05). During storage of the frying medium after frying the noodles, OGU and OG had higher oxidative stability with lower TBARS, PV, p-AnV, and Totox values than PO at room temperature for 12 days. Overall, using oleogel as frying media improved the physicochemical and nutritional properties of SDEW noodles. This finding could be beneficial for food industries to produce healthy fried food products for consumers.

## 1. Introduction

In food applications, edible oils are one of the most common frying mediums. However, these oils can have various adverse health effects depending on the amount of saturated and unsaturated fatty acid compositions. The most prevalent of these effects are chronic cardiovascular diseases [1]. Among various edible oils, palm oil is a predominant and popular one that is used widely by almost all countries around the world and has received much attention as a next-generation energy resource [2]. Thailand is the world’s third-largest producer of palm oil and is also one of the leading edible oil producers and exporters among the countries in Southeast Asia [3]. Most food companies use palm oil as the primary cooking medium for all foods, especially fried foods [4]. Instant noodle is an ultra-processed food that is very popular among many consumers because it is cheap, versatile, easy to carry, and easy to prepare at any time. Instant noodles are fried foods that are prepared using a wide range of ingredients, which vary in the different the regions where they are produced and consumed [5]. Thailand is also one of the key producers of salted duck eggs. These are typically cured with salted mud clay, which covers the duck eggs for 30 days [6]. Duck egg yolks are very popular in Chinese condiments and have a high economic value. However, due to their extreme saltiness (>4%), the egg whites are usually discarded due to lower or no economic value. Most recently, Lekjing and Venkatachalam [7] developed an innovative instant noodle product that utilized salted duck egg white (SDEW) as a unique ingredient, which effectively replaced the water and salt in the noodle composition and also increased the net protein content in the instant noodles.

Although instant noodles are very popular nowadays, the frying process impacts the consumer’s health, as it undergoes various undesirable changes including oxidation, the production of reactive oxygen species, and the deterioration of organoleptic and nutritional contents [5]. Therefore, the modification of oil into various other forms with better cooking properties is widely practiced, and recently the interest in oleogel usage has grown widely. This is mainly due to its unique thermo-reversible property, as it can undergo a transition from sol-gel at numerous times upon heating and cooling the system [2]. Oleogels are also known as organogels or structured oils, in which the continuous lipid phase is composed of edible oils which are generally condensed into a three-dimensional network by oleogelators. They have similar physical and chemical properties to solid fats [1]. Despite the differences in their structure and physical properties, oleogels are capable of replacing solid fats, which contain a high concentration of saturated and trans fats [8]. However, the application of oleogels in the food system is still not well tested due to the limitation in obtaining food-grade and inexpensive gelators [9]. There are various inexpensive gelators that have been tested with oil to produce oleogels. However, most of them were not used in food applications [9,10]. Recently, carnauba wax has been widely used as a gelator with various edible oils for food uses, and it is being regulated and certified as generally recognized as safe (GRAS) by the United States of America Food and Drug Administration and the Food and Agriculture Organization of the United Nations [4]. Carnauba wax is a vegetable wax that is mainly extracted from the leaves of carnauba palm trees. It is the hardest inert and stable wax with low solubility and a high melting point. It consists of esters (mainly cinnamic acid), aliphatic acids, hydrocarbons, terpenes, alcohols, and hydroxycarboxylic-free acids [5].

Carnauba wax is used in food applications as a food additive—specifically, as a gelator, encapsulation or emulsifying agent, glazing agent, acid regulator, and anticaking agent [11]. Among its various food applications, producing oleogel with carnauba wax is the most effective one, and it is normally made by mixing edible oil and carnauba wax at a certain high temperature (80–100 °C) [1]. Several studies reported that oleogel prepared with carnauba wax showed higher enthalpy of crystallization and strong stability against higher temperatures and oxidation as compared to beeswax [5,12,13]. Furthermore, a recent observation found that oleogel has been used as a fat replacer in many fried food products to reduce the oil uptake and saturated fats [9]. Although the traditionally processed oleogels have numerous benefits, a drawback persists regarding molecular rearrangement during ambient storage, releasing oil from the gel. On the other hand, ultrasonication (US) is one the most promising and multipurpose food processing techniques to be widely used for many decades. It is a safer and high-quality method that is mechanical and non-contaminant [14]. US is widely used in fat-rich ingredients to tailor the mechanical and functional properties. The US (≥20 kHz) process could help the dispersibility of the food composites, orient the molecules in the composite, and levitate and nanowire the microparticles across the full system of food without damage [10]. This research developed the palm oil oleogel using carnauba wax, processed it with ultrasonication, tested it as a fat replacement medium for SDEW instant fried noodles, and examined the various physicochemical properties of the instant noodles. It also examined the stability test of the tailored oleogels during storage at ambient temperature.

## 2. Results and Discussion

### 2.1. Color Characteristics

**Figure 1:**
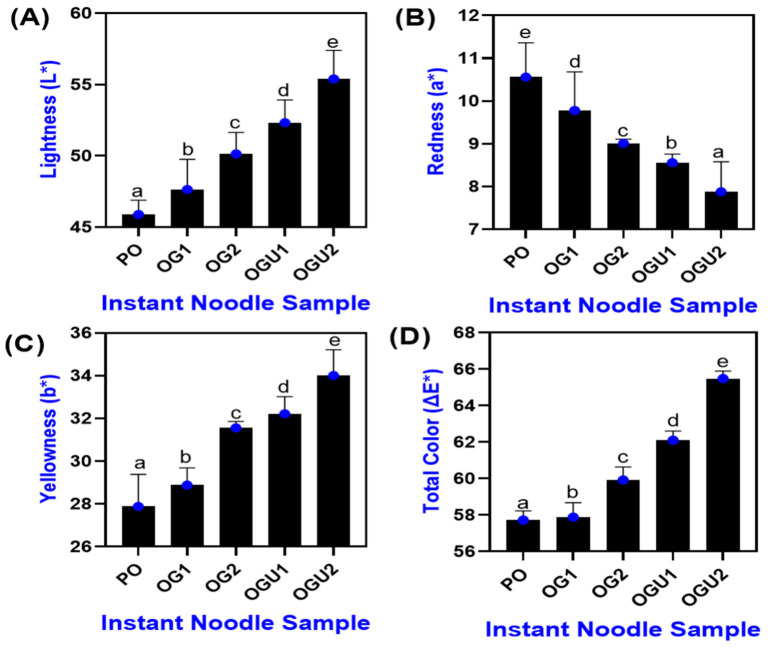
Color characteristics (L* (**A**), a* (**B**), b* (**C**) and ΔE* (**D**)) of palm oil, palm oil–carnauba wax oleogel and palm oil–carnauba wax oleogel with ultrasonication treatments. Note: PO represents palm oil; OG1 represents palm oil–carnauba wax (5 g/100 g); OG2 represents palm oil–carnauba wax (10 g/100 g); OGU1 represents palm oil–carnauba wax (5 g/100 g) homogenized with ultrasonication; and OGU2 represents palm oil–carnauba wax (10 g/100 g) homogenized with ultrasonication. The different alphabets (a–e) in the figures indicate significant differences.

The color characteristics of SDEW instant noodles that are fried in different oleogel frying mediums are shown in Figure 1A–D. Generally, in fried food products, the color characteristics play an important sensory role in the overall acceptability [9]. In general, the OGU-fried samples had the highest L*, b*, and lowest a* values, whereas the PO-fried samples showed the lowest L*, b*, and highest a* values (*p* < 0.05). A similar result was reported by Adrah et al. [1], in which the food product that was treated with canola oil–carnauba wax oleogel had an increased L* and decreased color intensity (a* and b*) when compared with canola oil-fried samples. The L* was found to be higher in OGU, followed by OG, when compared with PO (*p* < 0.05). This could be due to the different distribution and light reflectance of fat crystals in the oleogels that were used for frying [15]. Among the oleogel-fried noodles, the samples that were fried in oleogels (OG2 and OGU2) with a higher concentration of carnauba wax (10 g/100 g) showed a higher L* in comparison with the samples that were fried in oleogels (OG1 and OGU1) made with lower wax levels (5 g/100 g) (*p* < 0.05). This could be due to the increased concentration of carnauba wax in the oleogel, progressively increasing the number of fat crystals in the frying medium [16]. Thus, absorbed fat in the fried product enhances the light reflectance, which results in a higher L* value. Sonic waves from the ultrasonication application could have broken down the chemical bonds in the oleogel and facilitated the formation and uniform distribution of smaller fat crystals via the thermal, mechanical, mixing, and cavitation effects of sound waves [10]. This leads to the formation of more organized crystalline networks in oleogel. On the other hand, the PO-fried sample showed a dominant a*, whereas OGU2 had the lowest a* value (*p* < 0.05). It has been reported that the type of frying medium, the temperature, and the duration of frying greatly influence the overall appearance of the fried product [4]. Moreover, the a* decreased with augmenting the concentration of wax in the oleogel in both the OG and OGU samples (*p* < 0.05). A decreased level of the a* values was found in the SDEW instant noodles, particularly the ones that were fried using oleogels. This could be due to the lower occurrence of non-enzymatic browning reaction (Maillard reaction) during frying, as the noodles could be interfered or masked by the oleogels from the interaction between carbohydrate (source of carbonyl group) and proteins (source of the amino group) at a higher temperature [7]. In contrast, this phenomenon was not found in the samples that were fried using PO. In general, the higher redness of fried products is considered an undesirable quality characteristic that reduces consumer acceptability [4]. The b* value indicates yellowness, which is attributed due to the presence of flavonoids in the wheat flour [17]. The samples that were fried in PO had the lowest b* in comparison with those that were fried in oleogel (*p* < 0.05). Moreover, b* showed a similar trend with L* in all samples. OGU2 and OG2 had a higher b* than OGU1 and OG1 (*p* < 0.05). Carnauba wax was obtained from leaves of the Copernicia prunifera, widely available in the form of bright yellow flakes; thus, the addition of carnauba wax might have turned the oleogel towards a bright yellowish color, positively attributed to the color of noodles [11]. Natarajan and Ponnysamy [12] reported that ultrasonication-treated food products retained a high level of yellowness as compared with redness. Overall, noodles that were fried in ultrasound-assisted oleogel showed better color attributes, which increased overall consumer acceptance. The total color (ΔE*) value in the SDEW instant noodle samples was calculated using L*, a*, and b* values (Figure 1D), and the results showed that noodles fried using oleogels had slightly higher ΔE* than those fried with PO. Among the OG and OGU, the OGU samples retained more color values, and this increased with the increased carnauba wax concentration in the oleogel.

### 2.2. Cooking Properties

**Figure 2:**
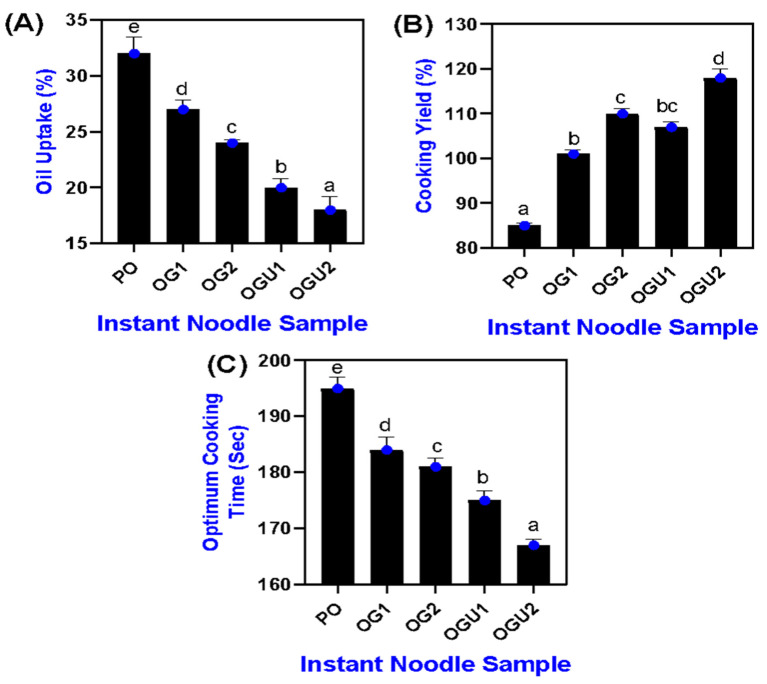
Cooking properties (oil uptake (**A**), cooking yield (**B**) and optimum cooking time (**C**)) of palm oil, palm oil–carnauba wax oleogel and palm oil–carnauba wax oleogel with ultrasonication treatments. Note: PO represents palm oil; OG1 represents palm oil–carnauba wax (5 g/100 g); OG2 represents palm oil–carnauba wax (10 g/100 g); OGU1 represents palm oil–carnauba wax (5 g/100 g) homogenized with ultrasonication; and OGU2 represents palm oil–carnauba wax (10 g/100 g) homogenized with ultrasonication. The different alphabets (a–e, bc) in the figures indicate significant differences.

Oil uptake is one of the critical parameters for determining the quality of instant noodles, and it is usually considered that lower levels of oil uptake in instant noodles results in the best quality [5]. The oil uptake of the SDEW instant noodles that are fried using different frying mediums is shown in Figure 2A. The present study explored whether SDEW instant noodles cooked with different frying mediums significantly influenced the oil uptake level in the noodle samples (*p* < 0.05). Oleogels considerably lowered the oil uptake in the noodle, as compared with PO, which held high-level oil in the noodles amongst all the samples. On the other hand, among the oleogels, the samples that were fried in OGU2 had the lowest oil uptake, followed by OGU1, OG2, and OG1, respectively (*p* < 0.05). Several studies have found that using oleogel as a frying medium could control the fried product’s oil uptake. The lower oil uptake of oleogel-fried samples is due to the higher viscosity of the oleogel [1]. It has been reported that the viscosity of the frying medium greatly influences the mass transfer of the food product that is subjected to frying [8]. Furthermore, the result indicated that oil uptake in the oleogel-fried instant noodle samples decreased when the concentrations of carnauba wax increased from 5 g/100 g to 10 g/100 g, *w*/*w* in the oleogel frying medium. This could be due to the self-aggregation of carnauba wax into a macro molecule through a non-covalent interaction, and forming a complex network that leads to the formation of a high viscous medium [18]. In addition, the ultrasonication treatment could increase the exposed hydrophobic groups in the oleogel, thus, controlling oil affinity towards the fried product and reducing the oil adsorption in fried products [10]. Sadiq et al. [19] reported that protein-rich food acts as a shield to cover its food surface, and this could also possibly control the affinity of hydrophobic elements between oil and food.

Cooking yield is the measurement that is taken for the sample after weight loss that occurs during cooking [20]. The cooking yield of SDEW instant noodles fried in different frying mediums is shown in Figure 2B. The cooking yield was found to be lower in oleogel-fried samples (OG and OGU) when compared to palm oil-fried (PO) samples (*p* < 0.05). This could be due to absorbed oleogel in fried products that might act as a barrier against moisture loss [8]. Moreover, the fried product’s cooking yield increased significantly with the concentration of oleogel in the frying medium. OG2 and OGU2 had a higher cooking yield than OG1 and OGU1, respectively (*p* > 0.05). The possible explanation is that moisture leaches out from the product while cooking [21]. After moisture loss, the void space remaining in the fried product is filled by fat. Therefore, cooking yield can be related to the fat adsorption of fried products [22]. A higher cooking yield generally represents lower oil adsorption of the finished product. In this study, the oleogel-fried sample recorded a higher cooking yield, and the results align with lower oil adsorption (see Figure 2A). The optimum cooking time for instant noodles in different frying mediums is given in Figure 2C. Overall, the optimum cooking time was found to be lower in all the samples due to the addition of SDEW. It has been reported that incorporating SDEW in instant noodles reduces the optimum cooking time and increases the cooking yield [7]. In addition, the lower gelatinization temperature could also decrease the optimum cooking time of the product [23]. The oleogel could further reduce the gelatinization effect of wheat flour, thus, decreasing the optimum processing time of the product [24]. The thermal conductivity is directly related to the optimum cooking time. Higher thermal conductivity of the sample leads to reduced optimal cooking time [21]. In this study, the optimum cooking time of OG and OGU samples was lower than that of the PO sample (*p* < 0.05), indicating that OG and OGU had the highest thermal conductivity. Among oleogel-fried samples, OGU2 had the lowest cooking time, followed by OGU1, OG2, and OG1, respectively (*p* < 0.05). Therefore, the higher optimum cooking time of PO-fried samples might cause a higher oil uptake, which insulates the noodles and creates a barrier against thermal conductivity and water affinity.

### 2.3. Textural Profile

**Figure 3:**
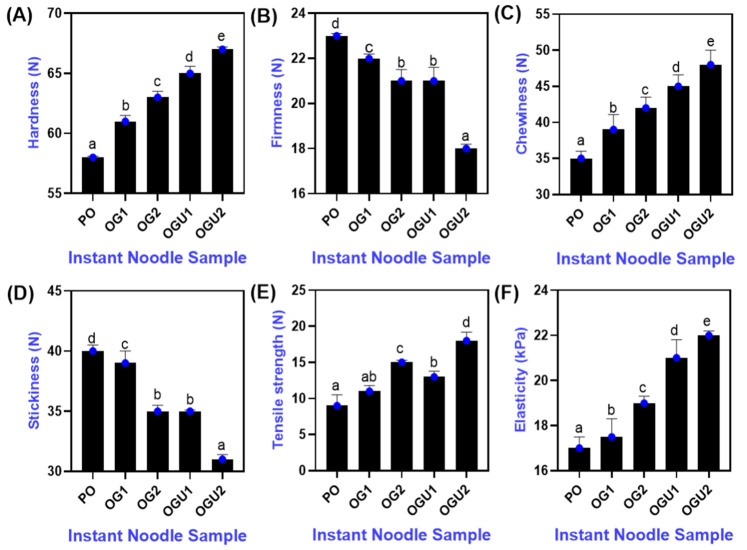
Textural profile (hardness (**A**), firmness (**B**), chewiness (**C**), stickiness (**D**), tensile strength (**E**) and elasticity (**F**)) of palm oil, palm oil–carnauba wax oleogel and palm oil–carnauba wax oleogel with ultrasonication treatments. Note: PO represents palm oil; OG1 represents palm oil–carnauba wax (5 g/100 g); OG2 represents palm oil–carnauba wax (10 g/100 g); OGU1 represents palm oil–carnauba wax (5 g/100 g) homogenized with ultrasonication; and OGU2 represents palm oil–carnauba wax (10 g/100 g) homogenized with ultrasonication. The different alphabets (a–e, ab) in the figures indicate significant differences.

The texture profiles of cooked SDEW instant noodles that are fried using different oleogel frying mediums are shown in Figure 3. Overall, this study showed a significant difference in the textural profiles of all the noodles samples that were fried using different frying mediums. Among the various frying mediums, the OG- and OGU-fried instant noodles showed a positive trend on the textural profile. A higher level of carnauba wax (10 g/100 g) and utilization of the ultrasonication process in the oleogel preparation (OGU) effectively influenced the noodle’s textural profile compared with the control (PO). Traditionally, the textural profile of SDEW instant noodles is mainly impacted by the influence of various ingredients used in the noodle compositions [7]. Sozer and Kaya [25] reported that noodles’ textural profile is one of the common indicators for estimating their degree of cooking. Hardness is typically the applied force required to compress the noodles [26]. This study showed that the frying medium significantly influenced the hardness of the SDEW instant noodles. Among the different frying mediums, a higher level of hardness was noticed in the samples that were fried in OGU than the OG- and PO-fried samples (*p* < 0.05). Furthermore, the addition of increased wax in the OG composition also positively affected the noodles’ hardness. Protein and starch contents are the predominant contributing factors to the product’s hardness [27]. During the cooking process, instant noodles could have gelatinized the starch content and generated a strong network with protein by interacting with SDEW proteins and wheat proteins [28]. The tensile strength of the SDEW instant noodles that were fried using OG and OGU also increased to a high level, as compared with PO. On the other hand, the tensile strength was found to be high in the samples that were fried in OGU, followed by OG and PO, respectively (*p* < 0.05). Among all the samples, the PO-fried sample registered the lowest level of tensile strength. The chewiness and elasticity were also found to be high in noodles using OG and OGU as frying mediums when compared to the PO. A higher concentration of carnauba wax in the oleogels and the ultrasonication process significantly increased the chewiness and elasticity of the samples. Chewiness and elasticity are primarily altered by noodles’ protein content, particularly wheat gluten [29]. Jung et al. [30] studied the incorporation of oleogels in bread compositions, and their results found that oleogels interfere with the gluten content and significantly increase the chewiness and hardness. Stickiness is the force required to remove the adhered sample from the probe [31]. This study observed a significant decrement in the firmness and stickiness of the samples that were fried in OG and OGU (*p* < 0.05). At the same time, the sample that was fried in PO had a higher level of stickiness and firmness. Oh and Lee [21] also observed a similar finding that adding oleogels in the noodles significantly decreased the firmness level compared to the samples without any oleogel treatments. Jang et al. [32] reported that thermal conductivity plays an essential role in altering the textural properties of noodles, and it increases significantly when cooked for a prolonged period under an increased moisture level. Overall, the SDEW instant noodles that were prepared in oleogel and ultrasonication-assisted oleogel were found to be harder, non-sticky, elastic, and more chewable than the samples that were fried in palm oil.

### 2.4. Microstructural Observation

**Figure 4:**
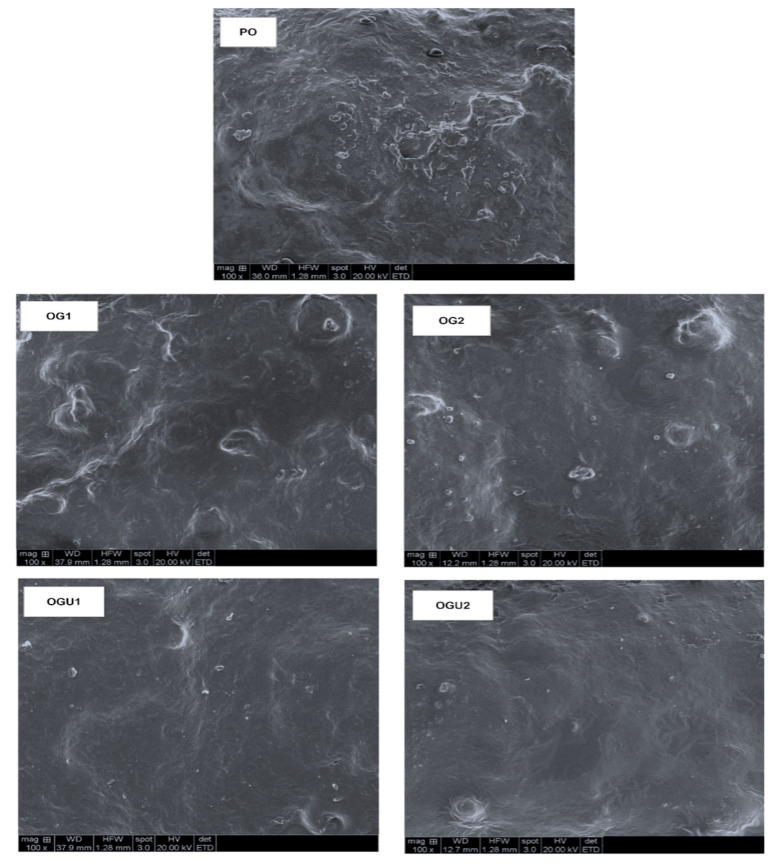
Microstructural observation of the surface of SDEW instant noodles that are fried using palm oil, palm oil–carnauba wax oleogel and palm oil–carnauba wax oleogel with ultrasonication treatments. Note: PO represents palm oil; OG1 represents palm oil–carnauba wax (5 g/100 g); OG2 represents palm oil–carnauba wax (10 g/100 g); OGU1 represents palm oil–carnauba wax (5 g/100 g) homogenized with ultrasonication; and OGU2 represents palm oil–carnauba wax (10 g/100 g) homogenized with ultrasonication.

The microstructural observation of SDEW instant noodles that are fried in different oleogel frying mediums is shown in Figure 4. Regardless of the frying medium used, the microstructural observation of the noodle sample’s surface section demonstrated significant morphological changes, particularly a rough and smooth surface. Overall, the SDEW instant noodles that were fried in oleogels (OG and OGU) had smoother surfaces with fewer bulges as compared to the palm oil-fried noodles with numerous rough, coarse, and bulged surfaces. It is generally believed that instant noodles are subjected to changes in morphological appearance, partly due to the ingredients and the frying medium in which they were cooked. The PO-fried samples had a denser and more complex rough surface, which might be due to the induced complex interaction between the protein content and gelatinized starch. This is in accordance with the study of Khatkar and Kaur [33]. Furthermore, the surface morphological differences of the noodle samples could also be associated with water vaporization during frying, which affects the structure of the product when the product’s water vaporization is increased and produces a coarse outer structure, thereby reducing the cooking yield [5]. In addition, a rough noodle surface (Figure 4) leads to increased oil absorption of the product [34], which can be seen in that the PO-fried noodles had a greater oil uptake and lowered the cooking yield as compared with the OG- and OGU-fried noodles (see Figure 2A,B). The gelling effect of oleogel could also be attributed to the uniform and smooth surface of the instant noodles, as they can rapidly solidify at ambient temperature as compared to PO, which is liquid and free flowable [9]. Xiao et al. [35] reported that, normally, plant lipids are flowable and easily exudate from the surface of the food products, causing an unfavorable morphological appearance on the food, whereas the oleogel has strong crystallization, which leads to minimal structural changes in the food products. The gelling effect of OG and OGU increased with the augmenting concentration of carnauba wax in the frying medium. The higher concentration of wax that was used in oleogel (OG2 and OGU2) had better morphological effects on the noodle samples as compared with OG1 and OGU1, respectively. When compared among the oleogel-fried products, the noodle samples that were fried with OGU had smoother surfaces as compared to the OG. This could be due to the gelation effect of the oleogels, which might be significantly better in the OGU samples. da Silva and Danthine [36] reported that the application of the sonication process in oleogel making could induce a faster gelling effect by facilitating rapid crystallization with smaller crystals and a more organized network. Li et al. [10] found that the ultrasonication increased the crystal clusters in the oleogel by generating more nucleation sites by acoustic cavitation effect.

### 2.5. Fatty Acid Profile

**Figure 5:**
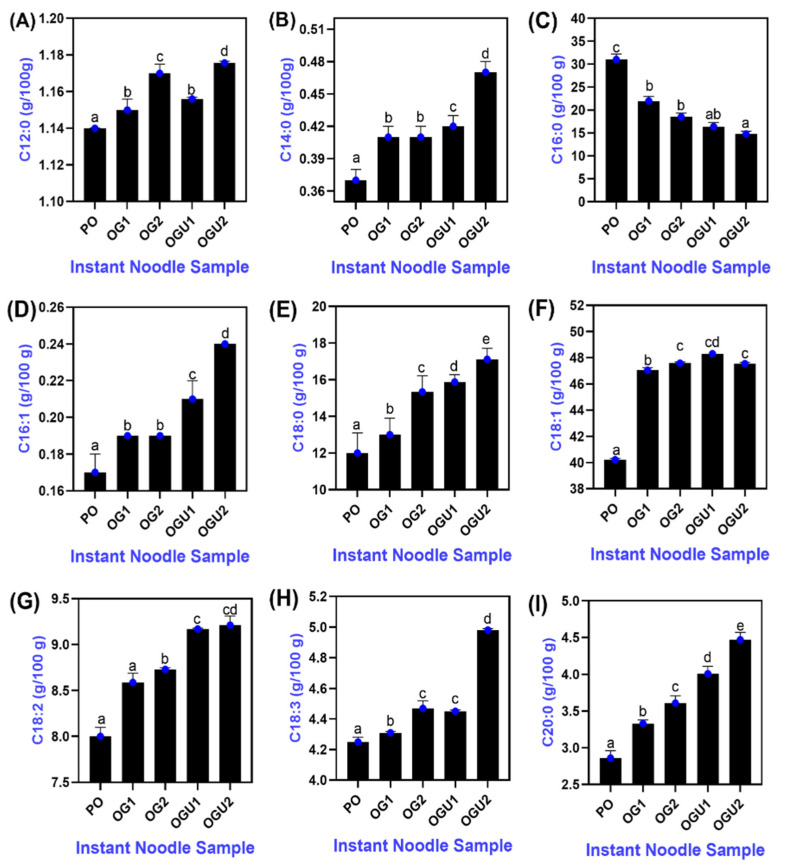
Fatty acid profile (**A**–**I**) of the surface of SDEW instant noodles that are fried using palm oil, palm oil–carnauba wax oleogel and palm oil–carnauba wax oleogel with ultrasonication treatments. Note: PO represents palm oil; OG1 represents palm oil–carnauba wax (5 g/100 g); OG2 represents palm oil–carnauba wax (10 g/100 g); OGU1 represents palm oil–carnauba wax (5 g/100 g) homogenized with ultrasonication; and OGU2 represents palm oil–carnauba wax (10 g/100 g) homogenized with ultrasonication. The different alphabets (a–e, ab, cd) in the figures indicate significant differences.

**Figure 6:**
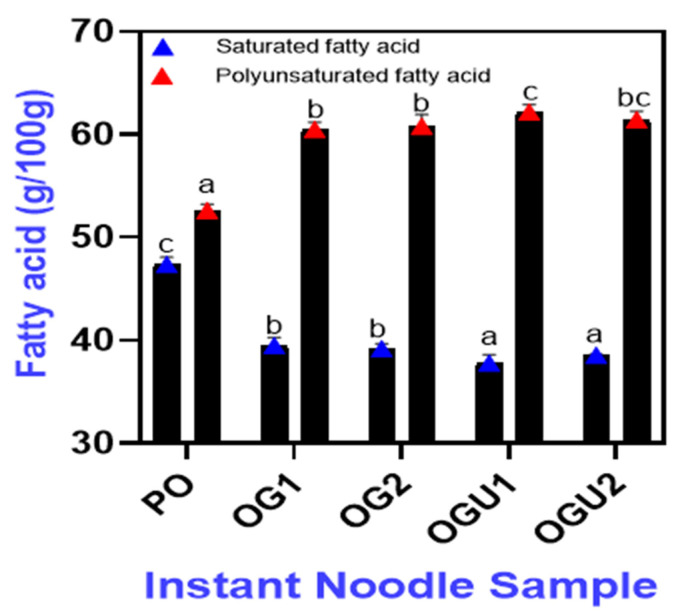
Cumulative level of saturated and polyunsaturated fatty acid content in the surface of SDEW instant noodles that are fried using palm oil, palm oil–carnauba wax oleogel and palm oil–carnauba wax oleogel with ultrasonication treatments. Note: PO represents palm oil; OG1 represents palm oil–carnauba wax (5 g/100 g); OG2 represents palm oil–carnauba wax (10 g/100 g); OGU1 represents palm oil–carnauba wax (5 g/100 g) homogenized with ultrasonication; and OGU2 represents palm oil–carnauba wax (10 g/100 g) homogenized with ultrasonication. The different alphabets (a–c, bc) in the figures indicate significant differences.

The fatty acid profiles of the SDEW instant noodles that are fried in different frying mediums are depicted in Figure 5. In total, nine fatty acids were observed in all the samples, which included saturated fatty acid (SFA) (palmitic acid; 16:0, lauric acid; C12:0, myristic acid; C140, arachidic acid; C20:0, stearic acid; C18:0); monounsaturated fatty acid (MUFA) (palmitoleic acid; C16:1, oleic acid; C18:1); and polyunsaturated fatty acid (PUFA) (cis-9,12-linolenic acid; C18:2, cis 6,9, 12 gamma-linolenic acids; C18:3), respectively. In general, unsaturated fatty acids (UFA) including MUFA and PUFA were noticed at a higher level than the SFA in all the fried SDEW noodle samples. The UFA to SFA content is of great importance in the nutrition of humans [37]. Mahmud et al. [38] documented that the higher content of UFA over SFA improved the overall nutritional quality of noodles that were incorporated with fish protein concentrate. In this study, the fortification of SDEW as a moisture and protein source in dough preparation might contribute to the high content of UFA in instant noodles. The salting process greatly alters the nutritional profile of duck eggs by reducing the triglycerides level and increasing the content of essential lipids [39]. On the other hand, cereal grains are a rich source of UFA, particularly linoleic and linolenic acid, which cannot be synthesized by humans due to the lack of desaturase enzymes [38]. Wheat flour in noodle formulation increases the UFA content of fried instant noodles [40]. Further, the addition of salted eggs reduces phospholipid and cholesterol levels in the food products, which are beneficial for human health [41]. Oleic acid (MUFA) was the most predominant fatty acid present in all samples, followed by palmitic acid (*p* < 0.05). Kaewmanee et al. [42] documented that oleic, palmitic, and linolenic acids are the most abundant fatty acids in salted duck eggs. The noodles that were fried in OG and OGU had a high content of SFA as compared to the PO-fried samples. Moreover, the SFA content of the noodle samples increased with the augmenting concentration of carnauba wax in the oleogels in both the OG and OGU samples, respectively (*p* < 0.05). Carnauba wax contains palmitic acid and stearic acid predominantly [43]. Therefore, the addition of carnauba wax in the oleogel preparations (OG and OGU) increased the SFA content of the noodles that were fried in oleogels. Among all the samples, the PUFA content was noticed to be higher in the samples that were fried in OGU, followed by OG, as compared to PO (Figure 6). PUFA, particularly linoleic acid, is the key component for the proper functioning of the nervous system [40]. In addition, the OGU-fried samples had high essential fatty acids as compared to the OG-fried samples in both concentrations. Ultrasonication greatly improves the functional properties of the oleogels, which has a positive effect on oleogel processed products [10]. The result revealed that noodles fried in oleogel and prepared using an ultrasonication process retained a high amount of fatty acid in the food products. Hence, the usage of oleogels processed with ultrasonication as the frying medium efficiently improved the nutritional quality of the SDEW instant noodles. Further, SDEW can be used as a nutritive-rich alternative egg product for the preparation of healthy foods for consumers.

### 2.6. Lipid Oxidation

**Figure 7:**
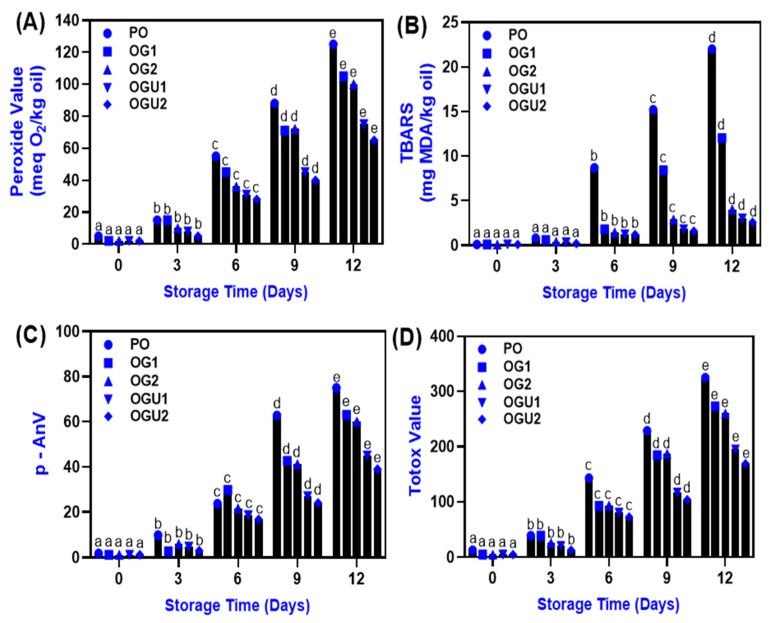
Changes in lipid oxidation (peroxide value (**A**), TBARS (**B**), p-AnV (**C**) and totox value (**D**)) during storage at ambient temperature in the different frying mediums that fried SDEW instant noodles. Note: PO represents palm oil; OG1 represents palm oil–carnauba wax (5 g/100 g); OG2 represents palm oil–carnauba wax (10 g/100 g); OGU1 represents palm oil–carnauba wax (5 g/100 g) homogenized with ultrasonication; and OGU2 represents palm oil–carnauba wax (10 g/100 g) homogenized with ultrasonication. The different alphabets (a–e) in the figures indicate significant differences.

The stability of oleogels and palm oil, which were used for frying the SDEW instant noodles, were tested for lipid oxidations (TBARS, peroxide value, p-anisidine value, and Totox value) under ambient storage for 12 days, and the results are depicted in Figure 7. Overall, the lipid oxidation results exhibited an increasing trend in all the samples (*p* < 0.05). Particularly, the PO sample was less stable and exhibited higher lipid oxidation as compared to the OG and OGU. Pérez-Álvarez et al. [44] observed that edible oil with higher or equal amounts of saturated and unsaturated fatty acid compositions is highly susceptible to lipid auto-oxidation. The TBARS results indicate a continuous accumulation of malondialdehyde (MDA) in all the tested samples during storage. Among all the samples, PO showed higher MDA, followed by the OG and OGU samples (Figure 7A). OGU exhibited better control against lipid oxidation as compared to OG, and different levels of carnauba wax incorporation to oleogels showed a significant control against the MDA. Park et al. [45] reported that TBARS has a high potential to react with a wide range of substances, particularly proteins. Thus, SDEW instant noodles are rich in proteins [7], and that might adversely affect the stability of the frying mediums after usage. Similarly, the peroxide value in the PO, OG, and OGU samples were significantly increased with augmenting storage time (*p* < 0.05). The OGU samples showed the least peroxide value as compared with the OG and PO samples (Figure 7B). A rapid increase in PV was noticed during the end of the storage period in all the samples. However, at the end of the storage period, PV was noticed to be lower in the oleogel than the palm oil due to the presence of a polymer structure in the oleogel [46]. An increase in the PV level in the samples indicates progressive lipid oxidation, which leads to the formation of hydroperoxides, a primary oxidation product [47]. The polymer structure in the oleogel provides a better network that resists the direct contact of oil with air [48]. The result was in agreement with Lim et al. [49], in which canola oil oleogel had lower oxidative products than canola oil during 60 days of storage. Moreover, it has been reported that polymeric oleogel has a tendency to extend oxidative stability due to its barrier properties against transition metals, free radicals, and pro-oxidants [50]. Therefore, the entrapment of oil in the carnauba wax provides oxidative stability to the palm oil over a prolonged storage time. Similarly, the p-AnV value increased gradually in all the samples during the storage period (Figure 7C). The OG and OGU samples had a lower accumulation of p-AnV during storage, whereas PO showed high p-AnV, and it steadily increased throughout the storage. Carnauba wax inclusion in the OG and OGU samples significantly controlled the accumulation of p-AnV. Furthermore, the controlling effect of carnauba wax against p-AnV increased with the increased wax concentrations. The p-anisidine indicates the accumulation of secondary lipid oxidative products, including aldehydes and ketones, in the samples during storage [51]. Lastly, the Totox value of PO, OG and OGU is shown in Figure 7D. According to the Totox value, prolonged storage significantly influenced the oxidation and reduction properties of the samples [52]. This is in accordance with Zaharan et al. [53]. Among the samples, PO was more susceptible to oxidoreduction reactions as compared with OG and OGU. The increased wax concentration in the oleogel and sonication process effectively reduced the increase in lipid oxidative products as compared with the other samples. Pan et al. [54] reported that the oleogels are a highly stable frying medium, as they significantly lowered the TBARS, PV, and p-AnV by entrapping the oil into a three-dimensional gel structure that retarded the oxidation caused by the air. Yu et al., 2022 [55] reported that the application of the sonication technique on the oleogel could induce a three-dimensional gel structure by promoting the enhanced hydrophobic interactions and hydrogen bonding between the edible oil and gelators, thus, improving various functionalities in the oleogel.

## 3. Conclusions

The present study explored the possibility of producing oleogels using palm oil, carnauba wax, and an ultrasonication process, and tested their efficiency and stability as an alternative frying medium against the conventional frying method using salted duck egg white fortified instant noodles. A significant improvement was observed in the physicochemical properties of oleogel-fried noodles when used with a higher concentration of carnauba wax and ultrasonication process. The noodle color, cooking yield, and optimum cooking time was significantly improved by frying in oleogels. Oil uptake was found to be higher in the noodles that were fried in palm oil, whereas a significant reduction in oil uptake was observed in oleogel-fried samples, especially the samples that were fried using oleogels assisted with the ultrasonication. Similarly, the textural profile of the instant noodles was also vastly improved by using oleogels as the frying medium. Noodles that were fried in oleogels and oleogels assisted with the ultrasonication process had a higher hardness, firmness, chewiness, tensile strength, and elasticity. A microstructural observation of the noodle samples that were fried in ultrasonication-assisted oleogels resulted in a uniform and smooth surface as compared with the conventional medium, which showed rough and bulgy surfaces. Further, noodles that were fried in oleogels exhibited superior nutritional quality in terms of the retention of fatty acids. The oxidative and storage stability of the oleogels was found to be very high as compared with the palm oil frying medium. The result showed that noodles fried in oleogels significantly controlled the oil uptake with improved textural attributes and better nutrient retention. Therefore, this study concludes that the application of oleogel (as well as ultrasonication-assisted oleogels) is an excellent frying medium for SDEW instant noodles and a good alternative to conventional frying medium.

## 4. Materials and Methods

### 4.1. Raw Material, Chemicals, and Reagents

The salted duck eggs (which have 4% salt) were purchased on the 20th day of the salting period from the commercial store in Chaiya district, Surat Thani Province, Thailand. After receiving them, the eggs were thoroughly cleaned using tap water and cracked open, and the egg whites were collected (SDEW) to use in the noodle compositions (see Section 2.3). Commercial grade refined palm oil and all other ingredients for the noodle compositions were purchased from the local supermarket in Surat Thani province. Carnauba wax flakes were purchased from DCMC corporation Co., Ltd., Bangkok, Thailand. All the solvents used in this study were acquired from ACI-Lab-Scan (Bangkok, Thailand), and all the chemicals were analytical grade and purchased from Sigma (St. Louis, MO, USA).

### 4.2. Oleogel Preparation

**Figure 8:**
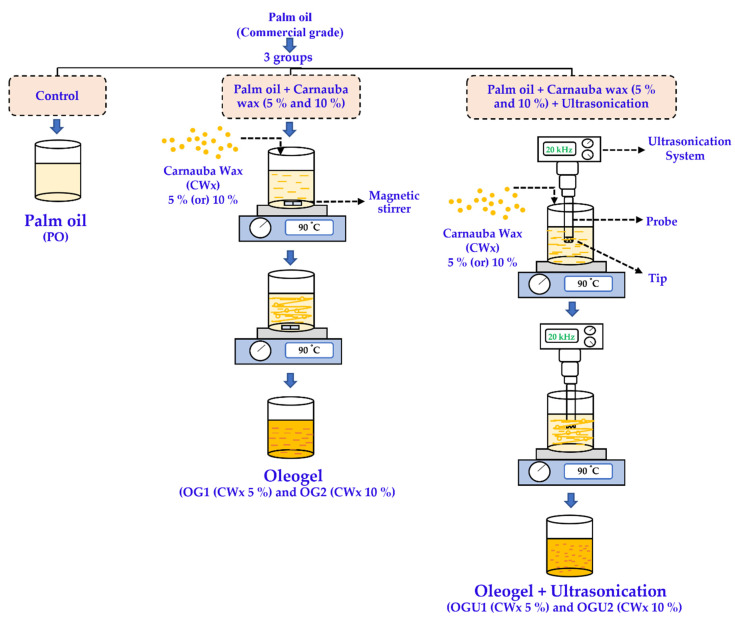
Pictorial reference of the preparation of the palm oil–carnauba wax oleogels and the ultrasonication assisted oleogels.

The oleogel as a frying medium was prepared using palm oil (base) and carnauba wax (gelator) with an ultrasonication process. Three different frying mediums were used, namely PO (commercial grade palm oil without any processing); OG (palm oil converted into oleogel using carnauba wax); and OGU (palm oil converted into oleogel using carnauba wax and further processed using ultrasonication). For OG, the carnauba wax at different concentrations (5% (OG1) and 10% (OG2)) was separately added into palm oil and mixed well using a temperature-controlled hot plate set at 90 °C and assisted with a magnetic stirrer for complete solubilization (wax melted less than a min, and the total oleogel process lasts for 5 min), followed by cooling at ambient temperature. For OGU, the carnauba wax at different concentrations (5% (OGU1) and 10% (OGU2)) were separately added into palm oil and mixed well using a temperature-controlled hot plate at 90 °C, which assisted with the ultrasonication process using a portable ultrasonic processor (Hielscher UP200Ht, Hielscher Ultrasound Technology, Teltow, Germany) that was equipped with a probe tip (40 mm) and processed at a constant 25 kHz for 10 min, followed by cooling at ambient temperature. All the prepared oleogels were stored in amber bottles at ambient temperature and used for frying the SDEW instant noodles within five days of their preparation. An infographic reference for producing oleogel is shown in Figure 8.

### 4.3. Preparation of SDEW Instant Noodle

SDEW instant noodle was prepared as tailored by Lekjing and Venkatachalam [7] with slight modifications. Firstly, the dough was prepared by mixing solid items including refined wheat flour (100%), SDEW (44%), sodium bicarbonate (0.26%), and ascorbic acid (0.40%) to the bowl of an electronic mixer (Cuzimate, RBSFOODMIXERPRO, Thailand). After mixing the solids, the SDEW (44%) was gradually added to the bowl and mixed to obtain the dough using the electronic mixer at a speed of 2000 rpm for 3 min at ambient temperature. After forming the dough, it was hand-kneaded for 5 min and placed in the refrigerator for 15 min, and then cut into small pieces and placed in a pasta roller to form noodle strands (1.5 mm width). Finally, the obtained strands were steamed at 90 °C for 2 min and then deep-fried using different frying mediums (from Section 4.2) at 150 °C for ~45 s. Then, the noodles were placed in the wired rack to cool down and allowed to drip off any excess oil. They were then packed in a sterile, airtight container and subjected to further analyses, as shown in Section 4.4.

### 4.4. Quality Analysis

#### 4.4.1. Color Characteristics

Color characteristics, including lightness (L*), redness (a*), and yellowness (b*), were recorded at random points on the fried SDEW instant noodle with the aid of the Hunter LAB colorimeter (Hunter Associates Laboratory, Inc., Reston, VA, USA). The total color characteristics of the noodles were calculated using the L*, a*, and b* values by following the equation proposed by Tiga et al. [56].

$$\Delta E^{*}=\sqrt{\Delta L^{*2}+\Delta a^{*2}+\Delta b^{*2}}$$

where ΔL*, Δa*, and Δb* represents the lightness, redness, and yellowness of the color characteristics of the SDEW instant noodles, respectively. ΔE* is the total color value.

#### 4.4.2. Cooking Properties

The oil uptake of the fried noodles was determined by following the method of AOAC [57]. Oil was extracted from the noodles via the solvent-extraction method using diethyl ether. Finally, the oil content was calculated from the percentage weight ratio of extracted oil to noodles. The cooking yield and optimum cooking time were determined as described by AACC [58]. For cooking yield, 5 g of instant noodles was boiled in 75 g of distilled water for 10 min with constant agitation. After draining the water from the noodles, the weight was taken before and immediately after cooking, whereas the optimum cooking time was determined by monitoring the core of the noodles during cooking; the final results were expressed in terms of a percentage.

#### 4.4.3. Texture Analysis

The texture profiles of cooked noodles were analyzed using a texture analyzer, as described by Lekjing and Venkatachalam [7]. The noodles were placed in a compression rig fitted with a 5 kg load cell. The textural attributes such as hardness, chewiness, and stickiness were measured from the obtained texture profile curve. The maximum peak at the first compression represents hardness, chewiness related to hardness, cohesiveness, and springiness, and stickiness is the negative area under the second peak. Firmness was expressed from the peak of the force–time graph. Further, the tensile strength and elasticity were recorded using force in tension mode and calculated using the following equation:

$$\mathrm{Tensile}\;\mathrm{Strength}\;\left(\mathrm{kPa}\right)=\frac{F}{A}$$

where F is the force (N) of firmness, A is the cross-sectional area of the noodle (m^2^).

$$\mathrm{Elasticity}\;\left(\mathrm{kPa}\right)=\frac{F}{T}\times \frac{L}{A}\times \frac{1}{v}$$

where F/t represents the initial slope of the force–time curve (N/S); L is the length of the noodle (0.015 m); A = the cross-section area of the noodle (m^2^); and v indicates the movement of the upper arm (0.003 m/s).

#### 4.4.4. Microstructure

Microstructural changes on the surface of the fried instant noodles were observed by using the method of Ahmad and Abozed [59] under scanning electron microscopy (SEM) using a JXA-840A, JOEL (Tokyo, Japan). The noodle was cut into small pieces and the surface section was fixed on an aluminum stub using double-sided adhesive tape. Then, the samples were sputter-coated with gold-palladium and followed by observing the image under SEM at 50× magnification.

#### 4.4.5. Fatty Acid Analysis

The fatty acid compositions of the fried noodles were analyzed using gas chromatography (Hewlett-Packard 6890, Agilent Technologies, Palo Alto, CA, USA) coupled with a flame ionization detector, as detailed by Lim et al. [5]. Triundecanoin, C11:0 (internal standard) in isooctane (1000 µg/mL) mixed with a lipid sample of the noodles and fatty acid methyl esters (FAME) was derivatized using boron trifluoride (14 g)/methanol (BF3-MeOH). Then, FAME was injected into the SP-2560 column (100 m × 0.25 mm ID, 0.20 mm film) (Supelco Bellefonte, PA, USA). The oven temperature was set at 100 °C for 4 min and then increased to 225 °C at a rate of 3 K/min and set for 20 min. The inlet and detector temperatures were maintained at 225 °C and 285 °C, respectively. The sample was injected in a 1:200 split ratio, and helium carrier gas with a flow rate of 0.75 mL/min was used. Peaks were identified by comparison with the authentic standard (Supelco FAME mix, Bellefonte, PA, USA), and the fatty acid content was expressed as g/100 g of oil.

#### 4.4.6. Oxidation Stability of Oleogels

The frying mediums, including PO, OG, and OGU after frying the SDEW instant noodles, were stored at room temperature for the oxidation stability analyses. The samples were taken randomly, and lipid oxidation was analyzed at an interval of 3 days throughout 12 days of storage.

##### Thiobarbituric Acid Reactive Substance (TBARS)

TBARS was analyzed as detailed by Rajasekaran et al. [47]. The oil (0.1 g) was mixed with 2.5 mL of TBA reagent. Then, the mixture was heated for 10 min at 95 °C, and absorbance was measured at 532 nm using a spectrophotometer (UV-160, Shimadzu, Kyoto, Japan). TBARS was determined using a standard curve of MDA (0–5 µm), and the result was expressed in mg MDA/kg oil sample.

##### Peroxide Value (PV)

The titration method was used to analyze PV [60]. The oil (0.1 g) was mixed with 25 mL of acetic acid/chloroform mixture at a 3:2 ratio. Then, 1 mL of saturated potassium iodide and 75 mL of distilled waste was added, and the mixture was kept in the dark for 5 min. Thereafter, the mixture was titrated with 0.01 N of sodium thiosulfate (Na_2_S_2_O_3_) after the addition of starch solution (1%) as an indicator. The endpoint of the titration is dark blue faded to a pink color.

$$\mathrm{PV}\;\left({\mathrm{meq}\;O}_{2}/\mathrm{kg}\;\mathrm{oil}\;\mathrm{sample}\right)=\frac{V\times M\times 1000}{W}$$

where V is the volume of Na_2_S_2_O_3_ (mL); M refers to the concentration of Na_2_S_2_O_3_ (N); and W is the weight of the sample (g).

##### p-Anisidine Value (p-AnV)

The oil (0.5 g) was dissolved in 1 mL of 0.5% of the p-anisidine solution. The absorbance was measured at 350 nm after 10 min using a spectrophotometer. p-AnV was computed as guided by Okpala [61].

$$p\text{-}\mathrm{AnV}=\frac{\left[25\times \left(1.2A2-A1\right)\right]}{M}$$

where A1 and A2 are the absorbances before and after the addition of p-anisidine solution, respectively, and M refers to the weight of the sample (g).

##### Totox Value

The Totox value represents the total oxidation, which can be calculated using PV and p-AnV by following the equation that was proposed by Sun and Waterhouse et al. [62].

Totox value=2PV+p−AnV

### 4.5. Statistics

All the analyses in this study were performed in triplicates, except for the textural analysis, which was carried out in ten replications, and the data are presented as mean ± standard deviation. The results were tested for significant differences by using a one-way analysis of variance (ANOVA) and Duncan’s multiple ranges as a post-hoc test with *p* < 0.05 as a standard level of significance. All the statistical analyses were performed using the Statistical Package for the Social Sciences (SPSS, SPSS Inc, Chicago, IL, USA).

## Data Availability

The data presented in this study are available on request from the corresponding author.
